# Accessory articulation of cervical vertebral transverse process: a rare case

**DOI:** 10.1259/bjrcr.20210119

**Published:** 2022-11-01

**Authors:** Sarmad Aslam, Iqrah Aslam, Jeffrey Tsang, Farris Latief, Adarsh Thatuskar, Naveen Rojed

**Affiliations:** 1 Department of Radiology, Lincoln County Hospital, Lincoln, UK; 2 King’s College London School of Medicine, London, UK; 3 Department of Medicine, Grantham & District Hospital, England, UK

## Abstract

There are several anatomical variants of the cervical vertebrae described in literature ranging from benign findings to those with varying clinical implications, including association with congenital diseases. We describe a case of an extremely rare anatomical variant of the cervical spine consisting of an accessory articulation of the cervical vertebrae C4 and C5 right transverse processes. The case is of a 35-year-old female who presented to primary care with 6-week history of intermittent, dull neck pain. An X-ray of the cervical spine was obtained which did not demonstrate radiological evidence of an acute fracture, dislocation, nor vertebral collapse. However, the X-ray revealed subtle multilevel anterior marginal osteophytic lipping. As a minor fragmented fracture or osteophytic growth could not be excluded; the patient proceeded to have a CT of the cervical spine. This revealed an accessory articulation of both the C4 and C5 right transverse processes, an extremely rare anatomical variant. Our comparison of both X-ray and CT imaging modality results highlighted the importance of utilising CT in identification in the accessory articulation anomaly, which aids in directing patient to appropriate care. Although similar cases may have been seen in clinical practice, to our knowledge, there are no previously documented C4-5 accessory articulations in the literature.

## Introduction

Anterior tubercle elongation of the cervical vertebra transverse processes is a rare congenital variation. There have been few anatomical variants of the cervical spine reported in the literature since it was first described in 1960 by Lapayowker.^1^ Within the English literature, our searched identified four previous case reports of such phenomena at the C5-6 vertebral level^2–5^ and only one report at the C6-7 level.^6^ Although it may have been seen previously, to our knowledge, we are the first to report an accessary articulation of both C4 and C5 right transverse processes.

## Case report

A 35-year-old female initially presented to primary care complaining of a 6-week history of intermittent, dull neck pain. Of note, she reported having sustained minor trauma to her neck 1 year ago. At the time, she did not present to hospital and thus no investigations were performed.

Upon the request of her GP, a routine X-ray of the cervical spine (C-spine) was performed with the aim to identify any possible fracture. This demonstrated no radiological evidence of an acute fracture, dislocation, nor vertebral collapse. However, instead it revealed subtle multilevel anterior marginal osteophytic lipping. As a minor fragmented fracture or osteophytic growth could not be definitively excluded, the patient was promptly referred for a CT scan to further investigate. The CT C-spine incidentally revealed an accessory articulation of both the C4 and C5 right transverse processes; an extremely rare anatomical variant. The patient was managed with simple analgesia.

## Discussion

There are various congenital anomalies of the cervical spine documented in the literature ranging from high ranging vertebral artery to foramen transversarium.^7^ Anatomical anomalies of the cervical spine are particularly difficult to recognise in the paediatric cohort due to rapid developmental anatomy. The anomalies in the paediatric cohort often coexist with skeletal dysplasias, connective tissue disorders and metabolic disorders. Examples of various cervical anomalies in children include basilar invagination, atlantoaxial instability, and atlantoaxial rotatory subluxation. Recognition of such anomalies is crucial to direct the patient to specialists for management of high-risk cervical anomalies and management of various genetic and metabolic syndromes.^8^ A study by Ankith et al assessed 930 CT scans from patients who presented to the emergency department due to cervical spine trauma or cervical myelopathy. 33.1% of CT scans demonstrated at least one congenital anomaly. Anomalies included platybasia, accessory OC articulation, vertebral fusion and cervical rib. Early recognition of such anomalies is important as it has impact on clinical course—particularly in the emergency setting.

The aberrant elongation of the anterior tubercle of the cervical transverse process is notably a rare finding. Bilreiro et al reported a case of unilateral accessory articular between fifth and sixth cervical spine in a symptomatic patient, noting four other cases in English literature describing similar findings.^5^ The case we report, to our best of knowledge, is the only one reporting an accessory articulation of both the C4 and C5 right transverse processes. Recognising this variant is clinically significant as it allows us to differentiate from other anomalies, such as osteophytes and tumour growths as well as suspected fracture fragments.

Knowledge of the embryological development of the vertebrae can help us to understand the causal origin of these congenital phenomena. The vertebral column is derived from migrating mesenchymal cells, which in turn proliferate and differentiate into chondroblasts. Hence, the vertebrae initially exist as a cartilaginous scaffold. The ossification of this cartilage model, whereby cartilage is converted into bone, occurs at three primary centres: one centrum and the two halves of the neural/vertebral arch. The transverse processes are lateral extensions of these neural/vertebral arches and in the cervical vertebrae, it is shaped such that there is both an anterior and posterior tubercle. In rare circumstances, elongation of the anterior tubercle of the cervical transverse process can occur beyond the normal margins and cause articulation.

**Figure 1.**
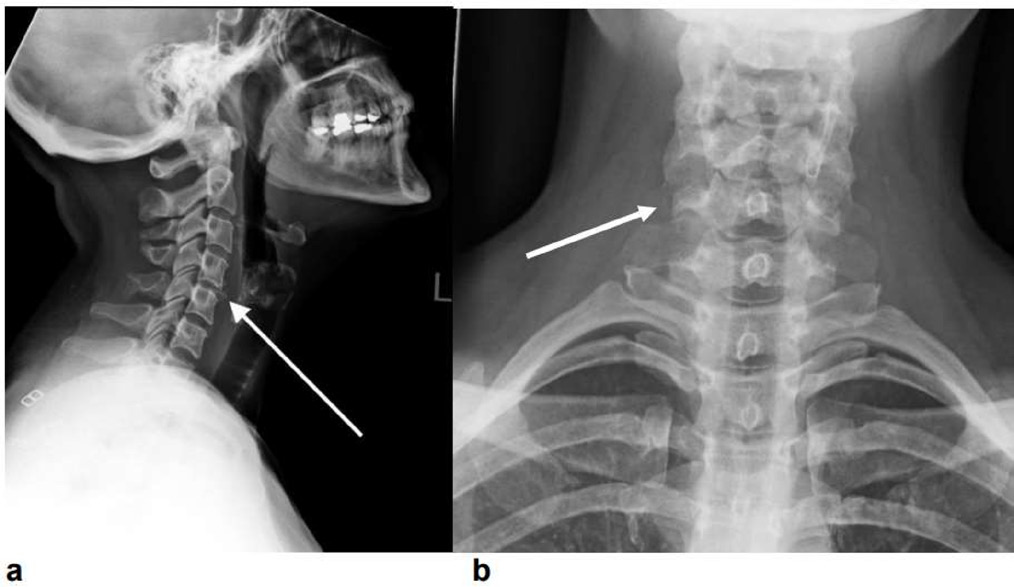
Lateral (**a**) and AP (**b**) plain film view of the C-spine demonstrating an anomaly at C4-C5 right transverse processes. AP,

On the lateral plain film view of the C-spine (Figure 1a), there is a subtle multilevel projection noted on the anterior margin of the vertebral bodies suggesting the possibility of cervical spine articulation at C4–C5. In addition, the vertebrae are normal in height and alignment and the intervertebral disc spaces appear relatively well preserved, with the pre-vertebral soft tissues within normal limits.

**Figure 2.**
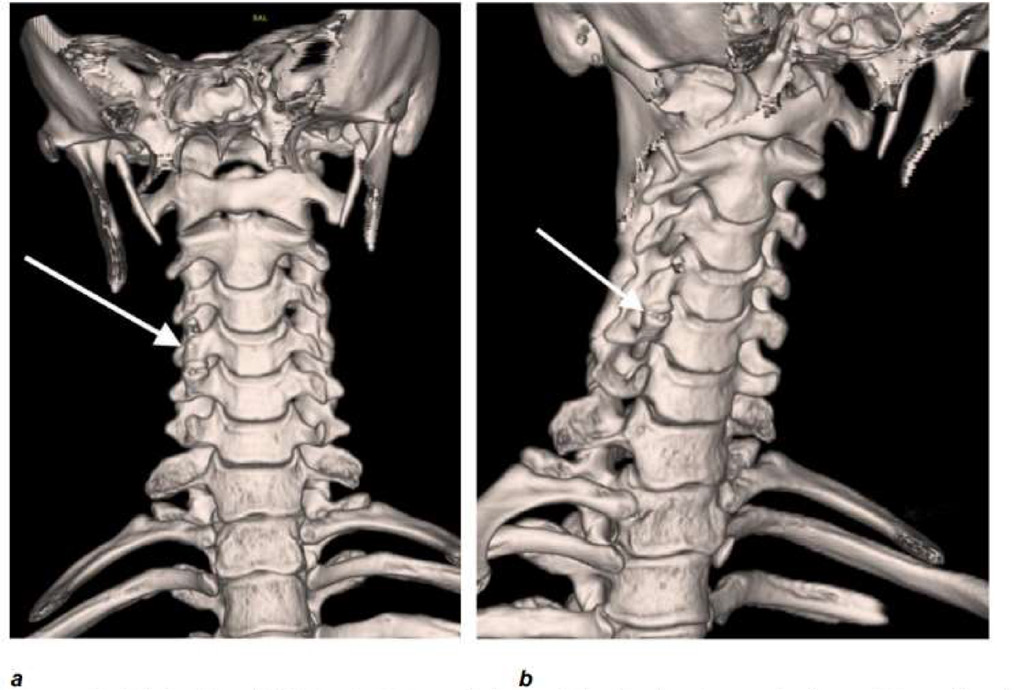
(a) Lateral; and (b) Anterior 3D model views of the C-spine demonstrating subtle multilevel anterior marginal projection noted, suggesting the possibility of cervical spine articulation at C4–C5.

**Figure 3.**
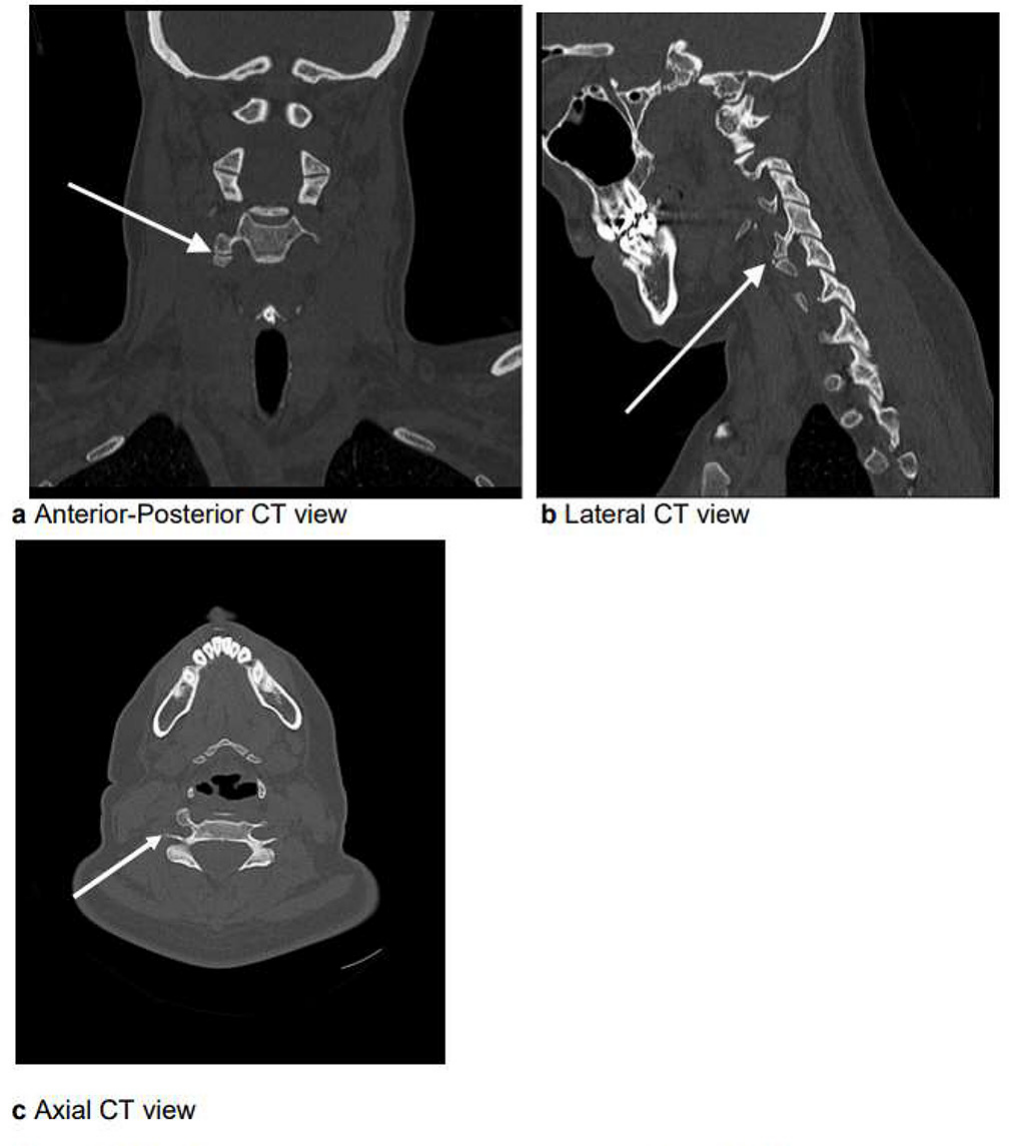
CT scan of C-spine showing an accessory articulation of C4 and C5 right transverse processes, as seen on the lateral cervical spine radiograph. Pathology associated with anomalous transverse processes is uncommon—with particular emphasis on the thoracic vertebrae. There is one case report in English literature with documentation of a T10 thoracic vertebrae with duplicated transverse process.^9^ The aetiology is unknown, but it is suspected that the anomaly is associated with a condition known as Klippel-Feil syndrome. Although, accessory articulation of the C4–C5 transverse processes is rare, there is a likeness to the more common variant of Bertolotti’s syndrome (also known as lumbosacral transitional vertebrae). Bertoltotti stated in 1917 that the presence of a variation of the fifth lumbar vertebrae with large transverse process, either articulated or fused with sacral basis or iliac crest can produce chronic, persistent lower back pain.^10^ Bertolotti syndrome is defined as congenital spinal anomalies with features of sacralization of the lowest lumbar segment or lumbarisation of the most superior sacral segment of the spine. Anatomical variation varies from L5 vertebrae with elongated transverse processes to S2 vertebral segment showing lumbarisation with formation of anomalous articulation to remainder of sacrum.^11^ The chronic back pain associated with Bertolotti syndrome is thought to be due to be due to arthritic changes occurring at the site of pseudoarthrosis. Hypothetically, the cervical spine accessory articulation site can be a location of pathology. The articulation could cause degenerative changes, nerve compression or muscular atrophy. The cervical transverse processes morphologically differ from the rest of the spine due to the foramen transversarium which contains the vertebral artery. It could be posed those patients with such articulation could be at risk of vertebral artery stenosis which leads to vertebrobasilar insufficiency syndrome.

Whilst there was no evidence of acute fracture, dislocation or vertebral collapse identified, it could not be completely excluded on plain film alone. This illustrates the advantage of CT compared to axial plain film imaging in delineating and detecting such subtle anomalies within a complex anatomical region. In Figure 2, her 3D models highlight the presence of an accessory articulation of the C4–C5 right transverse processes on her subsequent CT scan, Figure 3. The posterior elements are intact, with straightening of the cervical spine curvature and maintained vertebral heights seen. Reassuringly, again there is no fracture or subluxation identified.

## Conclusion

In summary, we report a very rare case of accessory articulation of the C4–C5 right transverse processes. Our comparison between the two imaging modalities, X-ray and CT, has highlighted the importance of CT in such complex cases—not only in delineation of anatomy and exclusion of other pathologies, but also in enabling us to deliver safe and appropriate care.

## Learning points

- Accessory articulations of spinal processes can present diagnostic uncertainty when relying on plain film images alone.
- CT scan of C-spine can be used to differentiate congenital anomalies and pathologies.
- It is important for requesting clinicians to provide good and accurate history when investigating spinal pathologies.
